# Morpho-functional effects of different universal dental adhesives on human gingival fibroblasts: an in vitro study

**DOI:** 10.1007/s10266-020-00569-x

**Published:** 2020-11-19

**Authors:** Stefano Pagano, Guido Lombardo, Egidia Costanzi, Stefania Balloni, Stefano Bruscoli, Sara Flamini, Maddalena Coniglio, Chiara Valenti, Stefano Cianetti, Lorella Marinucci

**Affiliations:** 1 School of Medicine, Department of Biomedical and Surgical Sciences, Odontostomatological University Centre: Chair Prof. Stefano Cianetti, University of Perugia, S. Andrea Delle Fratte, 06156 Perugia, Italy; 2 Department of Experimental Medicine, Section of Biosciences and Medical Embriology, University of Perugia, S. Andrea Delle Fratte, 06156 Perugia, Italy; 3 Department of Medicine, Section of Pharmacology, University of Perugia, S. Andrea Delle Fratte, 06156 Perugia, Italy

**Keywords:** Dental adhesive, Fibroblast, MTT, Apoptosis, Inflammation, ECM

## Abstract

To analyze the effects of four universal adhesives (Optibond Solo Plus—OB, Universal Bond—UB, Prime&Bond Active—PBA, FuturaBond M + —FB) on human gingival fibroblasts in terms of cytotoxicity, morphology and function. After in vitro exposure for up to 48 h, fibroblast viability was determined by the MTT assay determined, morphology by phase-contrast microscopy and migration by the scratch wound assay. Expression levels of IL1β, IL6, IL8, IL10, TNFα and VEGF genes were assessed by RT-PCR and their protein production by Western blot analysis. Apoptosis and cell cycle were analyzed by flow cytometry. OB and UB induced early morphological changes on fibroblasts (3 h) with extended cell death at 24 h/48 h. Gene expression of collagen type I and fibronectin increased fivefold compared with controls, elastin disappeared and elastase increased threefold, indicating gingival tissue tended to become fibrotic. Only UB and OB increased gene expression of inflammatory markers: IL1β at 3 and 48 h (up to about three times), IL6 and IL8 at 3 h (up to almost four times) which corresponded to the increase of the activated form NF-kB. All adhesives showed an effect on the functionality of fibroblasts with cytotoxic effect time and concentration dependent. Among all the OB and UB adhesives, they showed the greatest cell damage. The in-depth analysis of the effects of universal adhesives and possible functional effects represents an important information for the clinician towards choosing the most suitable adhesive system.

## Introduction

Universal adhesives were developed to solve clinical and practical problems in conservative dentistry allowing to obtain shortening [1] or fewer procedures and less manipulation during acid conditioning [2]. Also known as “multi-mode” adhesives, they may be used to self-etch on dentin or to etch and rinse on enamel, according to the type of caries and the clinician's choice [3, 4]. On the other hand, major disadvantages are a shallower enamel etching depth, greater discoloration of enamel margins and shorter adhesion duration than with a separate orthophosphoric acid step [5, 6].

Like standard adhesives, universals could, however, be associated with toxicity, i.e., immune or genotoxic alterations, or tissue reactions, such as inflammation or necrosis [7, 8]. Several studies associated adhesives with contact dermatitis, lichenoid reactions, sensitization reaction, parakeratosis or hyperkeratosis [9]. Many investigated their biocompatibility showing that 90% of residual TEGDMA and HEMA monomers were released within the first 24 h [10]. Released monomers could spread through dentine with the risk of hypersensitization and cytotoxicity [11]. Other studies highlighted alternative molecules, such as ethylene glycol and initiators (e.g., camphorquinone) as potential causes of cytotoxicity [12–15] but diverging results were due to both different methods of investigation and diverse parameters [1, 8, 16, 17]. Although no difference in cytotoxicity emerged in several studies [16, 18, 19], others observed etch and rinse [20–23] and self-etch adhesives [19, 24, 25] were associated with high cytotoxicity levels. Cytotoxic results were unclear in others [1, 17, 26, 27].

The different results in terms of cytotoxicity could be related to a different chemical composition. All adhesives contain monomers that may be hydrophilic (2-hydroxyethyl methacrylate (HEMA), 4-methacryloyloyloloethethy trimellitate anhydride (4-META) or hydrophobic like solvents, acetone or ethanol [28]. Universals have additional copolymers, silane [28] and carboxylate or phosphate monomers, such as methacryloyloxydecyl dihydrogen phosphate (MDP). MDP interacts with calcium and the precipitate occludes the tubules, helping to increase chemical adhesion [2]. Major changes in adhesion strategy over time might be another factor in their cytotoxicity. The total etch technique in the 5th generation was replaced by self-etching systems (6th and 7th generations) in which acid monomers partially demineralize the smear layer and underlying dentine [8].

Our preceding study showed that even though all adhesives in a series of universals were associated with a certain level of general toxicity, the behavior profiles were not the same over time and in varying dilutions [29]. Consequently, we postulated that factors other than cytotoxicity came into play in determining adhesive biocompatibility and turned our attention to the adhesive effects on fibroblasts as they are the predominant cell type in periodontal connective tissue [10, 30]. They are hypothesized to play a major role in modulating inflammatory process, as they activate, proliferate and secrete cytokines to counteract cell damage due to external stimuli, thus inducing inflammation [31]. Furthermore, external stimuli can alter normal fibroblast secretion of extracellular matrix (ECM) proteins.

The present investigation focused on early fibroblast responses to dental materials by analyzing the effects of four dental adhesives on morphology and function in terms of viability, apoptosis, the balance of pro- and anti-inflammatory markers and ECM molecule secretion and degradation.

Its first null hypothesis was that there are no significant differences in cytotoxicity in the four selected adhesives. The second null hypothesis was that contact between adhesives and fibroblasts does not determine any morpho-functional alteration of the gingival fibroblasts.

## Materials and methods

### Test materials

Starting from a previous study regarding cytotoxicity of dental adhesive on oral cell populations [29], four of them with self-etching and total etching techniques were examined: Optibond Solo Plus (OB; Kerr Corporation, Orange, United State), Universal Bond (UB; Tokuyama Corporation, Tokyo, Japan), Prime&Bond Active (PBA; Dentsply De Trey, Konstanz,Germany) and Futurabond M + (FB; Voco GmbH, Germany). Components, classification and manufacturer’s information are listed in Table 1.

**Table 1:** Composition and manufacturers of four dental adhesives

Adhesive (Code)	Composition	Manufacturer	Lot No.
Optibond Solo Plus (OB)	Methacrylate monomers (GPDMA, HEMA, Bis-GMA), inert mineral fillers (nanosilicate, disodium hexafluorosilicate), Ytterbium fluoride, photoinitiator, acetone, ethanol and water	Kerr Corporation, Orange, United State	6113662
Universal Bond (UB)	Methacrylate monomers (Bis-GMA, TEGDMA, HEMA), Hydrophilic monomers (MTU-6), silane coupling agent, peroxide, catalyst based of borate, acetone, isopropanol and purified water	Tokuyama Dental Corporation, Tokyo, Japan	008E57
Prime&Bond Active (PBA)	Methacrylate monomers (acrylate resin, multifunctional acrylate, bifunctional acrylate) Modified phosphoric acid, initiator, stabilizer, isopropanol and water	Dentsply De Trey, Konstanz, Germany	1706000157
FuturaBond M+ (FB)	Methacrylate monomers (Bis-GMA, UDMA), acid adhesive monomer, ethanol, water, catalyst	Voco GmbH, Germany	1724255

*Bis-GMA* bisphenol A (2-hydroxy propoxy) dimethacrylate, *GPDMA* glycerol phosphate dimetacrylate, *HEMA* 2-hydroxylethyl methacrylate, *MTU-6* thiouracil monomer, *TEGDMA* triethylene glycol dimethacrylate, *UDMA* urethane dimethacrylate

### Cell culture

Human gingival stroma fibroblasts BSCL138 (IZSLER, Brescia, Italy) were grown as monolayer cultures in sterile polystyrene T-75 flasks (Thermo Fisher Scientific, Waltham, MA USA) in a humidified incubator at 37 °C with 5% CO_2_ and twice-weekly changes of medium. The cultures were monitored under a phase-contrast Leitz inverted microscope.

The culture medium, Eagle’s Minimum Essential Medium (MEM, Thermo Fisher Scientific, Waltham, MA USA) was supplemented with 10% fetal bovine serum (FBS, Thermo Fisher Scientific, Waltham, MA USA), penicillin (100U/ml), streptomycin (100 mg/ml) and 25 μg/ml amphotericin B as anti-fungal agent (Thermo Fisher Scientific, Waltham, MA USA). Upon 80% confluence (logarithmic growth phase), cells were detached with a mixture of 0.25% trypsin and 0.02% ethylenediaminetetraacetic acid (EDTA). Cells were counted in a Countess Automated Cell Counter (Thermo Fisher Scientific, Waltham, MA USA) after 1:1 dilution in Trypan Blue Dye (10 μl of cells and 10 μl of Trypan Blue), and then plated as described below. All tests were performed between the seventh and ninth passage.

### Adhesive extract preparation

Dental adhesives (10 μl) were dropped centrally on the upper side of sterile glass discs (12 mm diameter × 0.15 mm depth, ExactaOptech Labcenter SpA, Modena, Italy), the solvent was evaporated with air spray without water and oil in according to the manufacturer’s instructions and samples were photocured (Bluephase^®^ G2, Ivoclar Vivadent AG, Schaan Liechtenstein). Light intensity was set to 300 mW/cm^2^ for OB ad PBA and 500 mW/cm^2^ for FB; the distance between bonding agents and the light-curing lamp tip was under 2 mm. Some dark custom-made spacers served to maintain an established distance between the light-curing tip and sample surface and to eliminate external irradiation sources. The polymerization times for adhesive materials were in accordance with the manufacturers’ instructions: 20 s for OB and 10 s for PBA and FB.

The adhesives on glass discs were topped with 1 ml of MEM (extract) containing 10% FBS, the anti-fungal agent (amphotericin B) and antibiotics (penicillin and streptomycin) for 24 h at 37 °C and 5% CO_2_. Extracts were filtered through 0.22 μm cellulose acetate filters (Merck Millipore, Germany) and serially diluted before use [29]. Collected extracts (culture medium + components leached from adhesives) were added to the cells undiluted or in serial dilutions. The pH of the extracts was evaluated: OB 7.3, UB 7.32, PBA 7.4, FB 7.38. All resulted in the growth pH range of a cell culture (7.2–7.4).

Extract dilutions and treatment times were as follows:

- MTT: dilutions 100%, 50%, 25%, 12.5%, 6.25%, 3.125%; timepoints 1, 3, 6, 24, 48, 72 h
- Cell morphology: dilution 100%; timepoints 1, 3, 48 h
- Scratch test: dilution 100%; timepoints 0, 18, 24, 48 h
- RT-PCR: dilution 100%; timepoints 1, 3, 24, 48 h
- Western blot analysis: dilution 100%; timepoints 1, 3, 24 h
- Apoptosis and cell cycle: dilution 100%; timepoint 24 h.

### Cytotoxicity assay (MTT)

Human gingival fibroblasts were seeded (10,000 cells/well) on optical clear 96-well flat bottom microtiter plates (Thermo Fisher Scientific, Waltham, MA USA) and incubated for 24 h a 37 °C in 5% CO_2_. Subsequently, the culture medium was discarded and replaced with 200 μl of diluted or undiluted extracts. Control groups were treated with fresh culture medium. Cell cultures were incubated for 1, 3, 6, 24, 48 and 72 h at 37 °C in 5% CO_2_. Cytotoxicity was assessed by a colorimetric assay measuring mitochondrial dehydrogenase activity. Reduction of the soluble tetrazolium salt, 3-[4,5-dimethyl-2-thiazolyl]-2–5-diphenyl-2H tetrazolium bromide (MTT, Sigma Chemical Co., St. Louis, MO, USA) to a formazan precipitate, causes a yellow-to purple colour change [32].

After treatment, 10 μl of MTT solution (5 mg/ml) was added to each well. Plates were covered and incubated for 4 h at 37 °C. MTT-derived formazan crystals were dissolved by adding 100 µl/well of dimethyl sulphoxide (DMSO, Sigma Chemical Co., St. Louis, MO) under gentle shaking for 30 min. Absorbance was measured at 570 nm using an automatic microplate spectrophotometer reader (Bio-Rad, Model 680 XR, CA).

According to ISO 10993-5 [33], fewer viable cells resulted in decreased mitochondrial enzyme activity (succinic dehydrogenase, SDH) which directly correlated with the amount of blue–violet formazan produced by the tetrazolium salt reduction. Absorbance values in the control group and the percentage of viable cells were compared. Cell viability was calculated according to the following formula using optical density (OD):

% cell viability = (OD ratio of test group/OD ratio of control group) × 100.

### Cell morphology

To determine the effects of extracts on cell morphology, human gingival fibroblasts were seeded at a density of 1 × 10^5^ cells/ml in 1.9 cm^2^ wells (Thermo Fisher Scientific, Waltham, MA USA) and maintained in MEM supplemented with 10% FBS, the anti-fungal agent (amphotericin B) and antibiotics (penicillin and streptomycin) until sub-confluence. The culture medium was then discarded and replaced with 1 ml of undiluted extracts. Control groups were treated with fresh culture medium. Cell cultures were incubated for another 1, 3 and 48 h at 37 °C in 5% CO_2_ before observation under a phase-contrast microscope (Nikon Eclipse MS100, Nikon Corporation, Tokyo, Japan).

### Scratch assay

To investigate fibroblast migration, cells were plated on 6-well flat bottom microtiter plates (Thermo Fisher Scientific, Waltham, MA USA) and grown in 2 ml growth medium. Once about 90% confluence was reached, medium was removed and a straight scratch along the monolayer was created in the centre of the well using a sterile P-200 pipette tip, as described elsewhere [34]. Cellular debris was gently removed with Dulbecco’s phosphate-buffered saline (PBS) and cultures were exposed to undiluted extracts. Images of wound closure were obtained at 0, 18, 24 and 48 h, using a conventional phase-contrast microscope (Olympus, Tokyo, Japan). Photographs were taken at 200× magnification to obtain cell behavior profiles of migration and morphology.

### RNA isolation and RT-PCR analysis

Human gingival fibroblasts were seeded (1 × 10^5^ cells/ml) in 6-well flat bottom microtiter plates (Thermo Fisher Scientific, Waltham, MA USA). After reaching confluence, cells were treated with undiluted adhesive extracts or fresh medium (control groups) for 1, 3 and 48 h to assess gene expression of inflammatory markers and ECM proteins and for 24 h to analyze apoptosis and cell cycle genes.

Total RNA was isolated as described elsewhere [35]. Briefly, RNA from control and treated fibroblasts was isolated using a total RNA purification kit (Thermo Fisher Scientific, Waltham, MA USA), and quantified by reading the optical density at 260 nm on a BioPhotometer (Eppendorf, Milano, Italia). Then, 1 μg of total RNA was subjected to reverse transcription (RT) in a final volume of 20 μl using ABM (Richmond, Canada). Real-time PCR was performed using 2 μl of cDNA from the RT reaction. The primer sequences of each gene are listed in Table 2. Primers were designed with PERL primer software using NCBI EntrezGene reference sequences as template and synthesized by Thermo Fisher Scientific. Real-time PCR was carried out in an Mx3000P cycler (Stratagene, Amsterdam, Netherlands) using FAM for detection and ROX as a reference dye. One-step PCR was performed in 25 ml of Brilliant SYBR(r) Green QPCR Master Mix (Stratagene, Amsterdam, Netherlands) according to the manufacturer’s instructions. At each annealing step, product formation was monitored with the fluorescent double-stranded DNA-binding dye SYBR(r) Green. The relative expression level of the housekeeping gene glyceraldehyde-3-phosphate dehydrogenase (GAPDH) was used to normalize marker gene expression in each sample. Immediately after PCR, a melting curve was undertaken by raising the incubation temperature from 55° to 95 °C to confirm amplification specificity. The expression was determined using the threshold cycle (Ct), and relative expression levels were calculated via the 2^−∆∆Ct^ method. All values were computed with the MxPro QPCR Software (Stratagene, Amsterdam, Netherlands).

**Table 2:** Primer sequences used for RT-PCR analysis

mRNA	Sequences (5′–3′)	Product (bp)
GAPDH	Fw: TGGTATCGTGGAAGGACTCATGAC Rv: ATGCCAGTGAGCTTCCCGTTCAGC	188
Fibronectin	Fw: TTTTGAGAGCTGATGACAGACA Rv: GCTCTTAATGGCAGAGAGGA	150
Collagen type I	Fw: GTGAGACAGGCGAACAGG Rv: GACCAGCAGGCACAGAGG	129
MMP1	Fw: TACACGCCAGATTTGCCAAG Rv: ATGAGCAAGATTTCCTCCAG	189
MMP2	Fw: CTGGAGAACTAGAGAAGGAC Rv: GAGGAGTACAGTCAGCATCT	147
MMP12	Fw: TGCTGATGACATACGTGGCA Rv: AGGATTTGGCAAGCGTTGG	69
Elastin	Fw: CTGGAATTGGAGGCATCG Rv: ACCTGGGACAACTGGAAT	200
VEGF	Fw: TGCTGTCTTGGGTGCATTGG Rv: GGTGCAGCCTGGGACCACT	72
IL-1β	Fw: GGACCTGGACCTCTGCCCTCTGG Rv: GCCTGCCTGAAGCCCTTGCTGTAG	80
IL-6	Fw: CAGAACAGATTTGAGAGTAGTGA Rv: CGCAGAATGAGATGAGTTGT	200
IL-8	Fw: GACATACTCCAAACCTTTCCA Rv: AACTTCTCCACAACCCTCT	162
Bcl-2	Fw: AGATGTCCAGCCAGCTGCACCTGAC Rv: AGATAGGCACCCAGGGTGATGCAAGCT	366
p53	Fw: GGACCTGATTTCCTTACTG Rv: TGAATCTGAGGCATAACTG	248
p21	Fw: TGGAGACTCTCAGGGTCGAA Rv: GACTGCAGGCTTCCTGTGG	118
p16	Fw: CCCAACGCACCGAATAGTTA Rv: CACCAGCGTGTCCAGGAA	173

### Protein extraction and western blot analysis

Human gingival fibroblasts were seeded (1 × 10^5^ cells/ml) in 6-well flat bottom microtiter plates (Thermo Fisher Scientific, Waltham, MA USA) and, after reaching confluence, treated with undiluted adhesive extracts or fresh medium (control group) for 1, 3 and 24 h. After treatment, fibroblasts were washed twice with ice-cold PBS and detached with trypsin/EDTA solution as described above. They were then covered with MEM, centrifuged at 1200*g* for 5 min at 4 °C and washed twice with PBS. Total proteins were extracted by lysing the cells with radioimmunoprecipitation assay (RIPA) lysis buffer (HiMedia Laboratories, Einhausen, Germany) supplemented with phosphatase inhibitor cocktails and EDTA 1X. Lysates were left for 30 min on ice, vortexed every 10 min and stored at − 20 °C overnight. Finally, samples were centrifuged at 12,000*g* for 10 min at 4 °C and the supernatants (total protein) were collected [36].

Protein concentrations in the cytosolic extracts were quantified using the Bio-Rad assay; 30 μg per lane was loaded on 12% SDS-PAGE and transferred on to nitrocellulose membranes. To reduce nonspecific binding, membranes were blocked with 5% (w/v) no-fat dried milk in T-TBS (TBS containing 0.1% Tween-20) for 1 h at room temperature. After blocking, membranes were incubated overnight at 4 °C under gentle agitation with each primary antibody: rabbit anti P-NF-kB–p65(Ser536) polyclonal antibody (1:250) in 5% milk, rabbit anti NF-kB—p65 polyclonal antibody (1:1000) in BSA, or rabbit anti-cathepsin B polyclonal antibody (1:750) in BSA. All antibodies were purchased from Elabscience (Houston, Texas, USA). Membranes were stripped and re-probed with mouse anti-β-actin mAb antibody (1:5000) as a loading control. After washing twice in T-TBS, membranes were incubated with horseradish peroxidase (HPR)-labeled anti-rabbit or anti-mouse (both 1:5000) secondary antibodies for 1 h at room temperature. Immunoreactive proteins were detected using the enhanced chemiluminescence system (ECL, Amersham Pharmacia, Milan, Italy) and quantified with an image analyzer (ChemiDoc, Biorad, California, USA).

### Apoptosis and cell cycle analysis

Apoptosis and cell cycle analysis were assessed by flow cytometry as previously described [37]. Briefly, controls and fibroblasts were harvested after 24 h, re-suspended in 0.5 ml hypo tonic propidium iodide (PI) solution (50 µg/ml propidium iodide in 0.1% sodium citrate plus 0.1% Triton X-100) and analyzed by flow cytometry using Coulter Epics XL-MCL Flow Cytometer (Beckman Coulter). Data were analyzed using FlowJo software (TreeStar).

### Statistical analysis

Figures report the mean ± SD (standard deviation) of three independent experiments performed in quintuplicate for each dental adhesive. One-way analysis of variance (ANOVA) was performed using GraphPad Prism 5.01 software (Prism, CA, USA). *p* values of < 0.05 were considered statistically significant.

## Results

### Cytotoxicity assay (MTT)

All undiluted adhesive extracts were associated with time-dependent SDH activity. It increased over the short term s (1, 3, 6 h) but was reduced long term (from 24 to 72 h). The drop was most marked in FB and UB extracts at 72 h (37% and 49%, respectively).

**Fig. 1:**
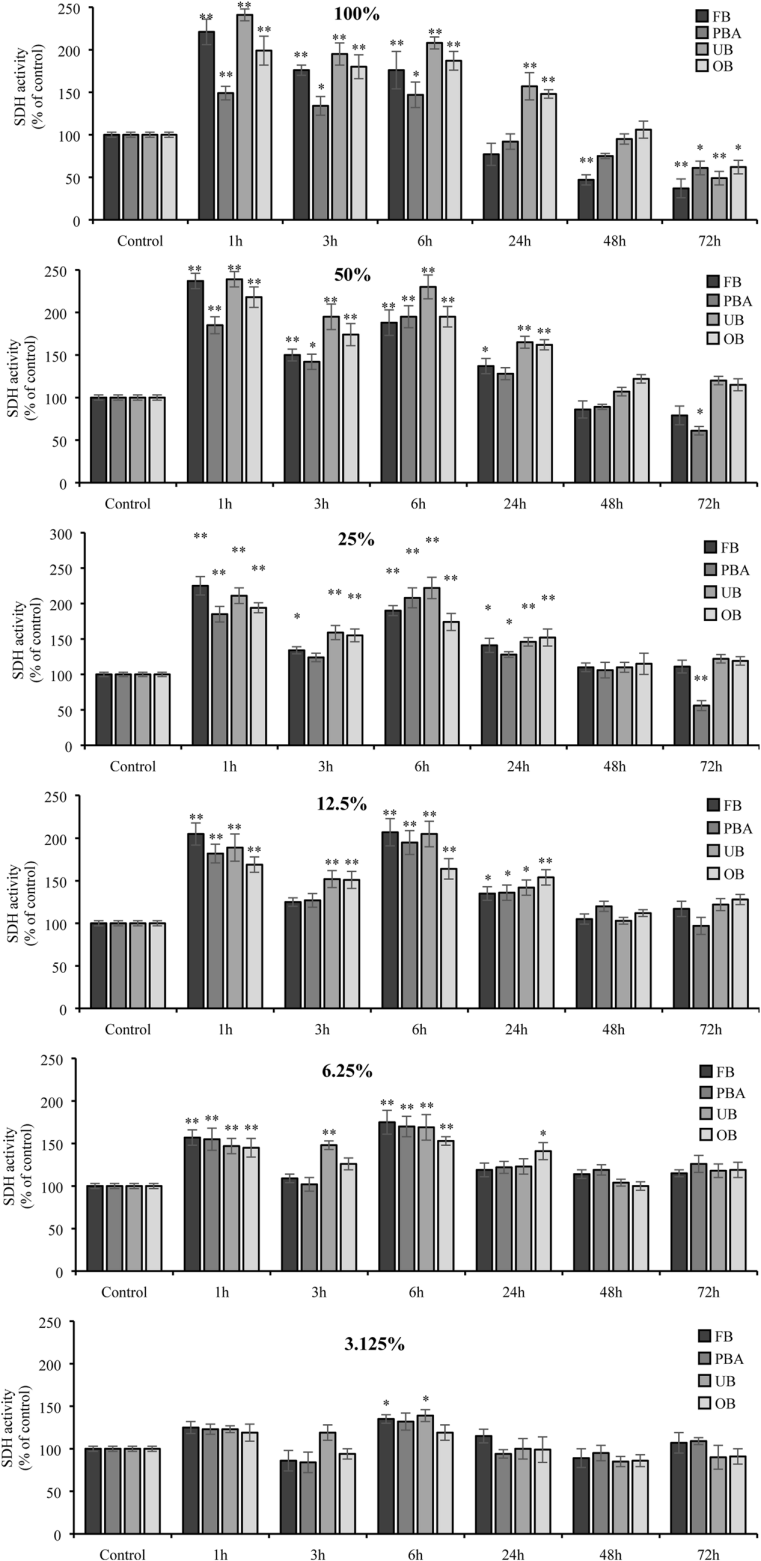
Effects of dental adhesive extracts (diluted and undiluted) on human gingival fibroblasts using the MTT assay. The results for each extract are expressed as the percentage of SDH activity compared with the control (100%). The values represent the mean ± SD of three independent experiments performed in quintuplicate for each sample. Differences vs. control: *p < 0.05; **p < 0.001

As extracts were diluted, short-term stimulation and long-term inhibition of cell viability were less marked (*p* = ns) (Fig. 1).

### Cell morphology

**Fig. 2:**
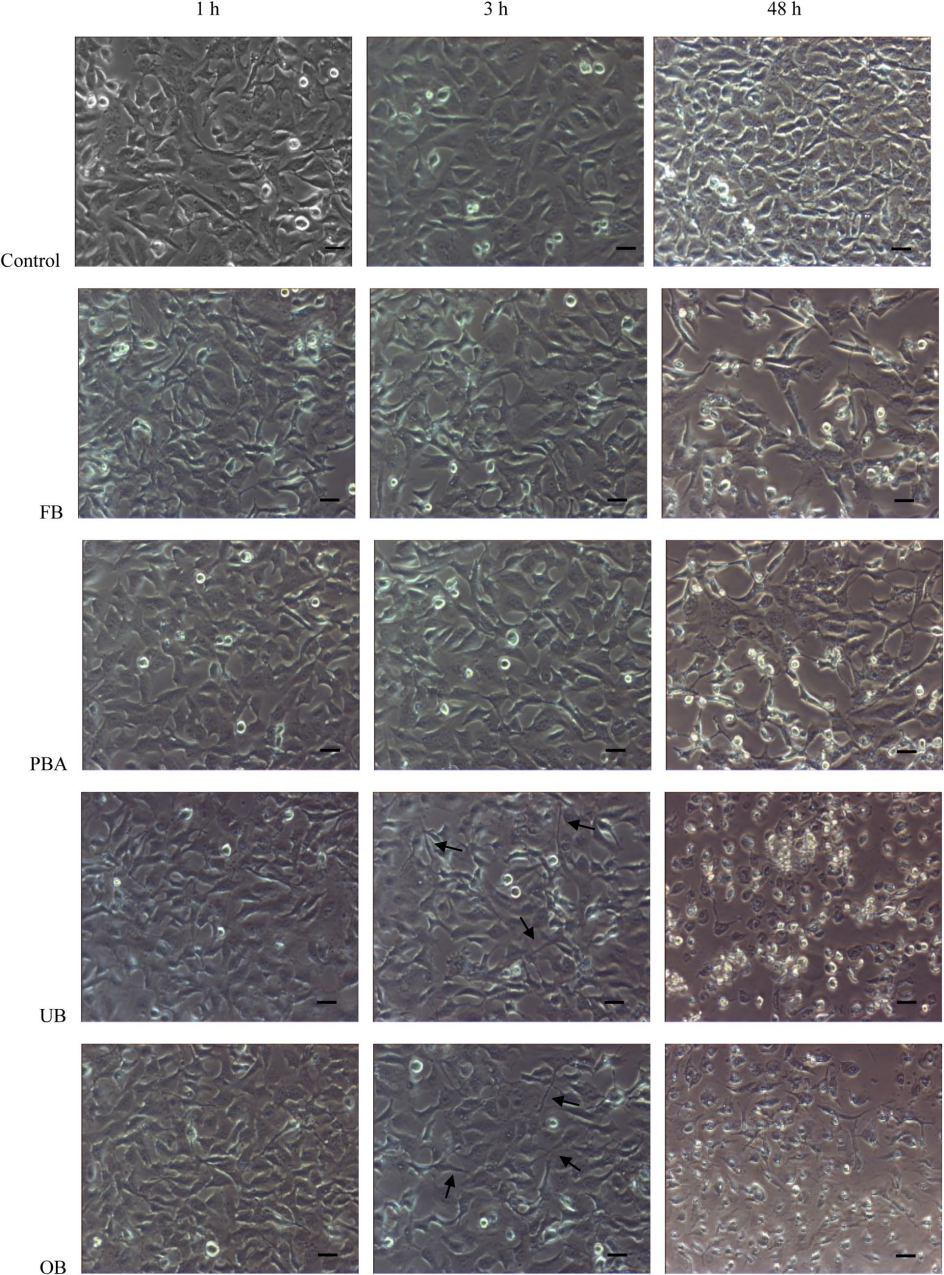
Time-dependent effects of adhesive extracts on fibroblast morphology. Phase-contrast micrographs of untreated human gingival fibroblasts (control) or fibroblasts exposed to undiluted extracts for 1, 3 and 48 h. Arrows indicate spindle cells with threadlike extensions (Bar = 10 μm)

Under a phase-contrast microscope, controls always (1, 3, 48 h) displayed a continuous monolayer of viable fusiform-shaped cells. After 1 h, elongate morphology was unchanged in all four extracts. After 3 h, wide intercellular spaces (low density cellular sheet) were observed and cells showed a prevalently spindle or irregular shape, with less defined borders and many threadlike extensions. After 48 h, cell numbers dropped and numerous detachable, round cells were detected, indicating that adhesive extracts had a toxic effect. All these changes were more marked in cells that were treated with OB and UB extracts (Fig. 2).

### Scratch assay

**Fig. 3:**
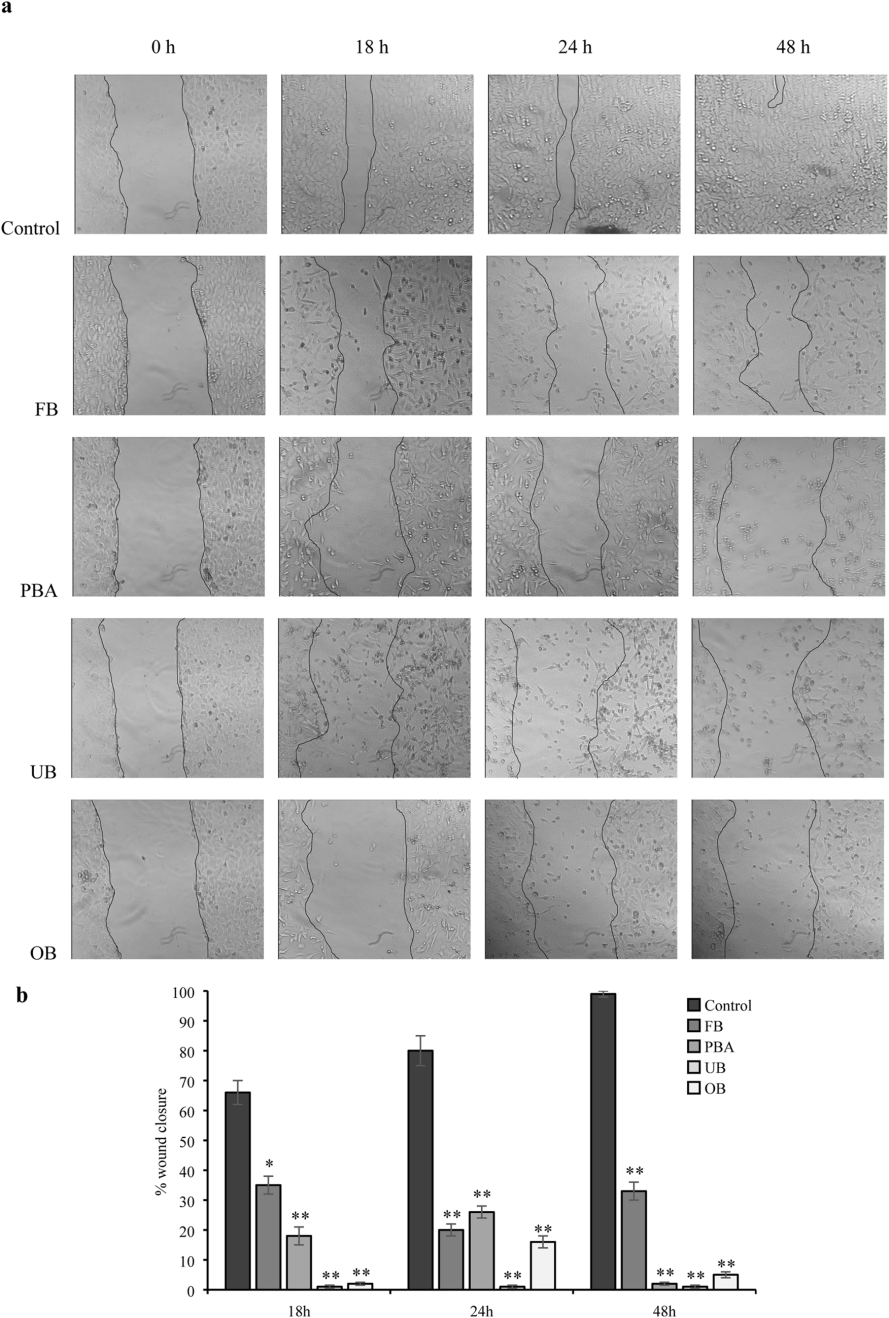
Effect of undiluted adhesive extracts on cell migration in the wound-healing migration assay. **a** Representative phase-contrast images of the wounds were taken at 0, 18, 24 and 48 h (200× magnification). **b** Quantification of the percentage of closed wound area calculated by tracing the border of the wound using ImageJ software. Data represent the mean ± SD of three independent experiments. Differences vs. control: *p < 0.05; **p < 0.001

With all dental extracts, the scratch was not still closed at 48 h, unlike controls. After 18 h only FB and PBA were associated with scratch closing but narrowing was less than in controls and the scratch was still visible at 24 h. At all timepoints, cells in all extract samples were multiform and longer than controls, with gradually enlarging intercellular spaces (Fig. 3).

### Gene expression of inflammatory markers

To test the impact of the adhesive extracts on inflammatory processes, we analyzed the expression levels of IL-1β, IL-6, IL-8, IL-10, TNFα and VEGF genes by RT-PCR.

FB: IL1β upregulation was observed only at 48 h, with no change at 1 h and 3 h. Both IL6 and IL8 were downregulated at 1 h, while IL8 showed an increasing trend at 3 h and at 48 h. IL6 was upregulated at 3 h, dropping to baseline at 48 h.

PBA: IL1β displayed no significant changes. IL-6 and IL8 were downregulated at all timepoints.

OB: IL 1β expression was significantly upregulated at 3 h and 48 h. IL-6 and IL-8 were significantly upregulated (more than twofold) at 3 h and then gradually downregulated to baseline at 48 h.

UB: IL1β expression was upregulated at 3 h and 48 h. IL-6 and IL-8 expression was significantly upregulated at 3 h. They then downregulated to baseline at 48 h.

**Fig. 4:**
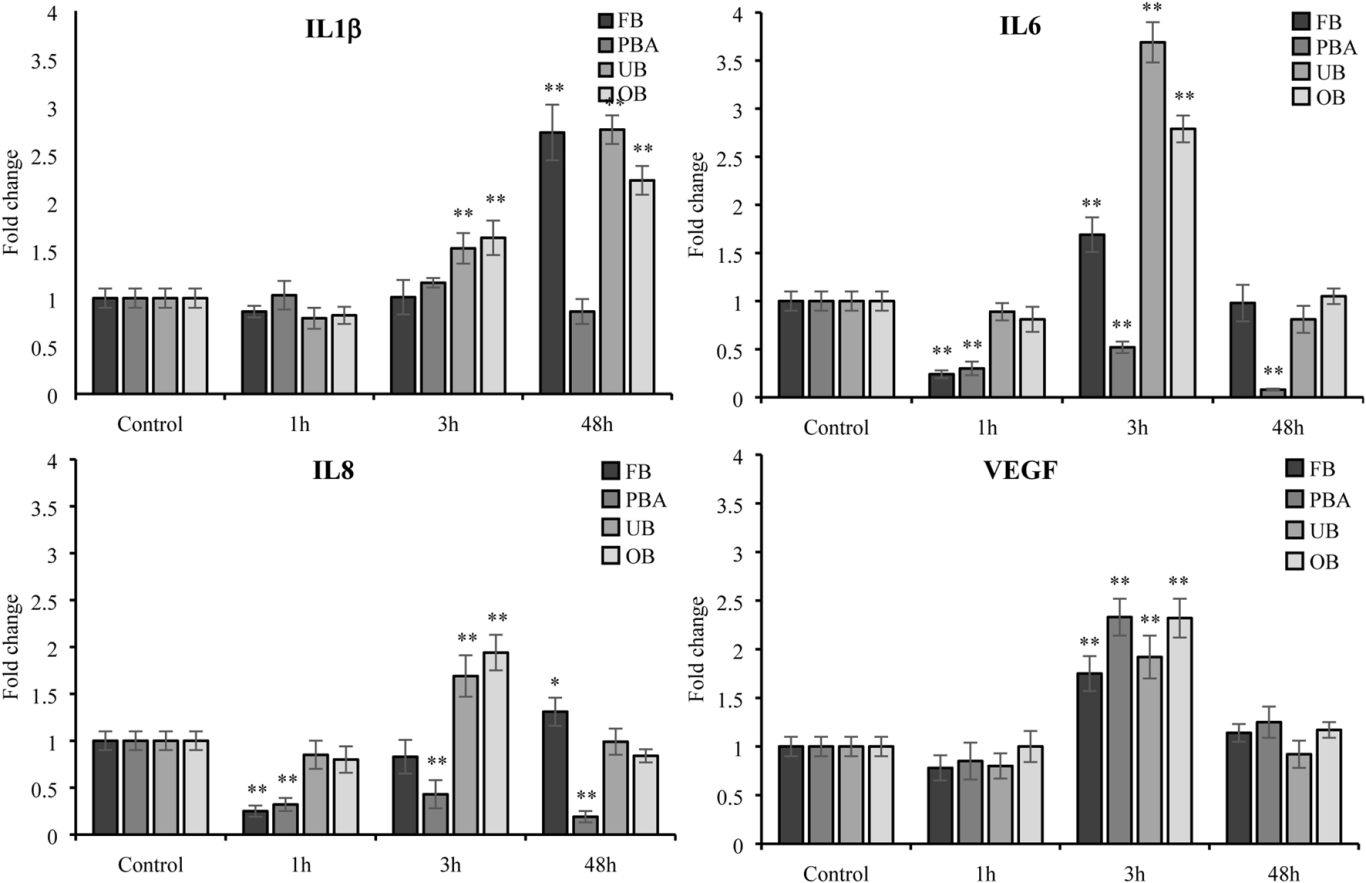
Effect of undiluted adhesive extracts on gene expression of IL1β, IL6, IL8 and VEGF evaluated by RT-PCR at 1, 3 and 48 h. The results for each extract are expressed as fold-change in GAPDH normalized mRNA values. The values represent the mean ± SD of three independent experiments performed in triplicate for each sample. Differences vs. control: *p < 0.05; **p < 0.001

No adhesive changed IL-10 and TNFα expression at any timepoint (data not shown). All adhesives upregulated VEGF expression at 3 h (Fig. 4).

### Gene expression of ECM proteins

PBA upregulated collagen I and MMP1 collagenase expression significantly (*p* < 0.05 and *p* < 0.001 respectively) after 1 h. OB significantly increased only collagen I transcription after 1 h (*p* < 0.001). All adhesive extracts significantly increased collagen I and MMP1 collagenase expression from 3 h onwards. Significance was more marked at 48 h (*p* < 0.001).

FB and PBA stimulated fibronectin transcription at 1 h (*p* < 0.001), returning to baseline at 3 h and persisting there at 48 h. UB and OB upregulated fibronectin significantly at 3 h, reaching more marked significance at 48 h (*p* < 0.001). No adhesive changed MMP2 mRNA (gelatinase) expression significantly at any timepoint.

**Fig. 5:**
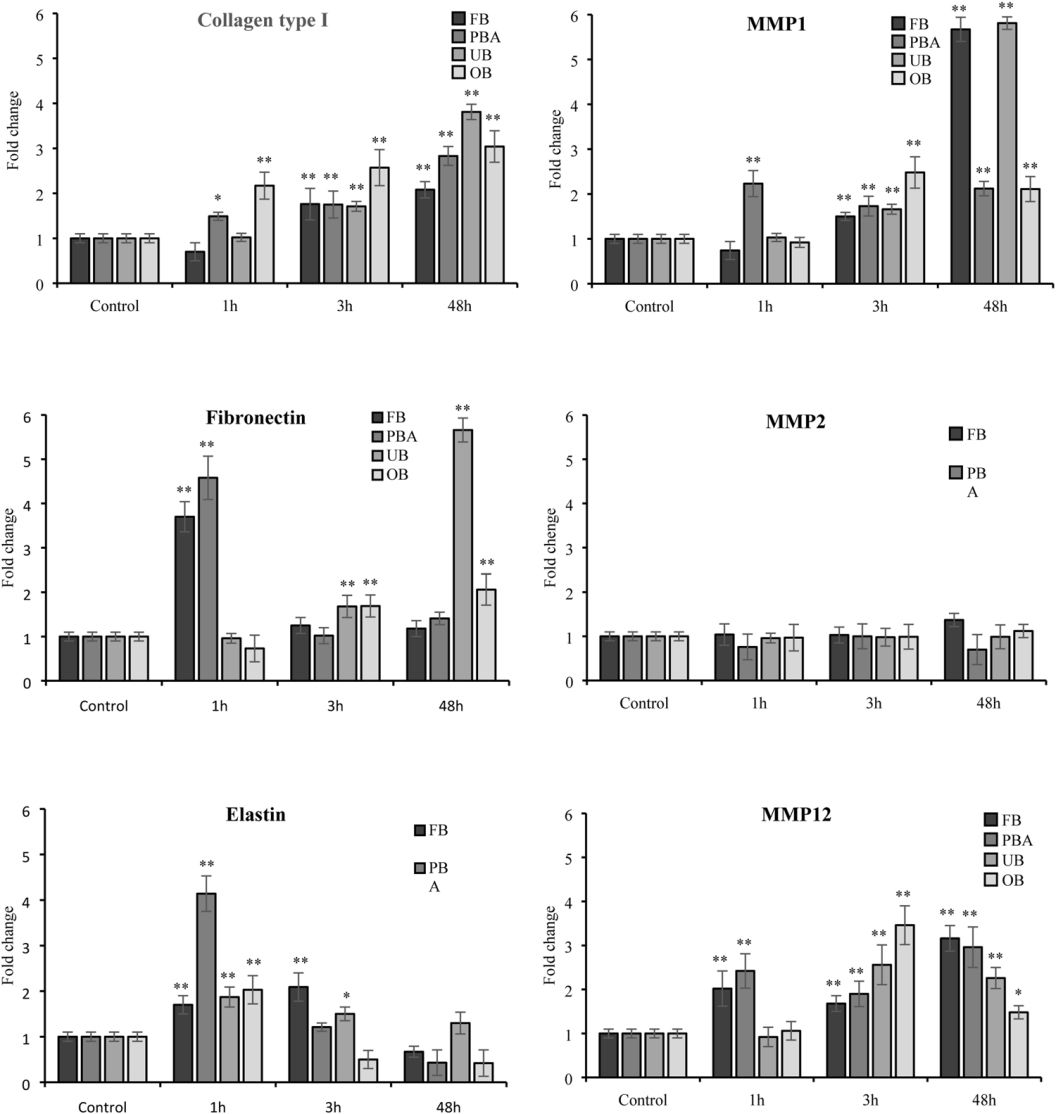
Effect of undiluted adhesive extracts on gene expression of collagen I, fibronectin, elastin, MMP1, MMP2, and MMP12 evaluated by RT-PCR at 1, 3 and 48 h. The results for each extract are expressed as fold-change in GAPDH normalized mRNA values. The values represent the mean ± SD of three independent experiments performed in triplicate for each sample. Differences vs. control: *p < 0.05; **p < 0.001

All extracts upregulated elastin expression at 1 h (*p* < 0.001). Only FB and UB maintained upregulation at 3 h (*p* < 0.001 and *p* < 0.05 respectively). Elastin expression returned to baseline at 48 h. All extracts upregulated MMP12 expression (elastase) at 3 h and 48 h. FB and PBA significantly increased MMP12 transcription after 1 h (*p* < 0.001) (Fig. 5).

### Western blot analysis

Western blot analysis investigated the effects of four adhesive extracts on cathepsin B and transcription factor NF-kB-p65 expression and activation (p-NF-kB-p65), both involved in the inflammatory pathway, after 1, 3 and 24 h.

**Fig. 6:**
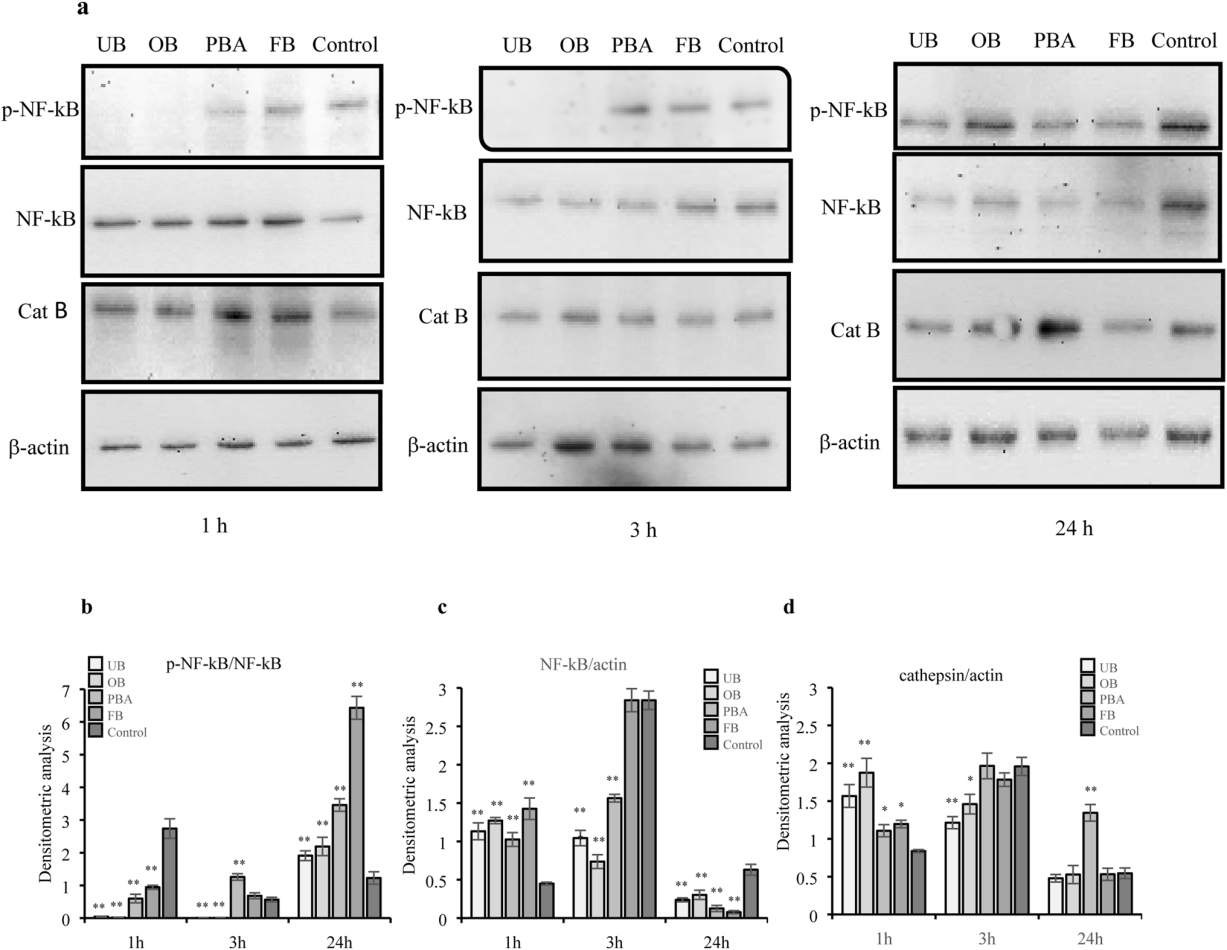
Effect of undiluted adhesive extracts on protein expression of p-NF-kB-p65, NF-kB-p65 and cathepsin B evaluated by Western blot analysis at 1, 3 and 24 h. Western blots are representative of three independent experiments. **a** Immunoblot of p-NF-kB-p65, NF-kB-p65, cathepsin B and actin. Densitometric analysis of proteins bands of p-NF-kB-p65 (**b**), NF-kB-p65 (**c**) and cathepsin B (**d**). The values represent the mean ± SD of three independent experiments. Differences vs. control: *p < 0.05; **p < 0.001

One hour after treatment, UB and OB inhibited p-NF-kB expression compared with controls. PBA and FB inhibited expression more weakly (Fig. 6a, b). After 3 h, UB and OB continued inhibition of p-NF-kB expression. Only PBA significantly increased expression twofold compared with control (Fig. 6b). After 24 h, all adhesives significantly upregulated p-NF-kB expression. Compared with controls, upregulation ranged from twofold with UB and OB to threefold for PBA and sevenfold for FB (Fig. 6b).

After 1 h, all adhesives significantly increased NF-kB expression (Fig. 6c). After 3 h, the increase was more marked but dropped below control levels at 24 h (Fig. 6c).

After 1 h, all adhesives significantly increased cathepsin B expression compared with controls. The rise was twofold with UB and OB (Fig. 6d). At 3 h, UB and OB significantly decreased cathepsin B expression compared to 1-h levels and controls. PBA and FB increased expression (Fig. 6d). At 24 h, all adhesives except PBA significantly decreased cathepsin B expression (Fig. 6d).

### Apoptosis and cell cycle

**Fig. 7:**
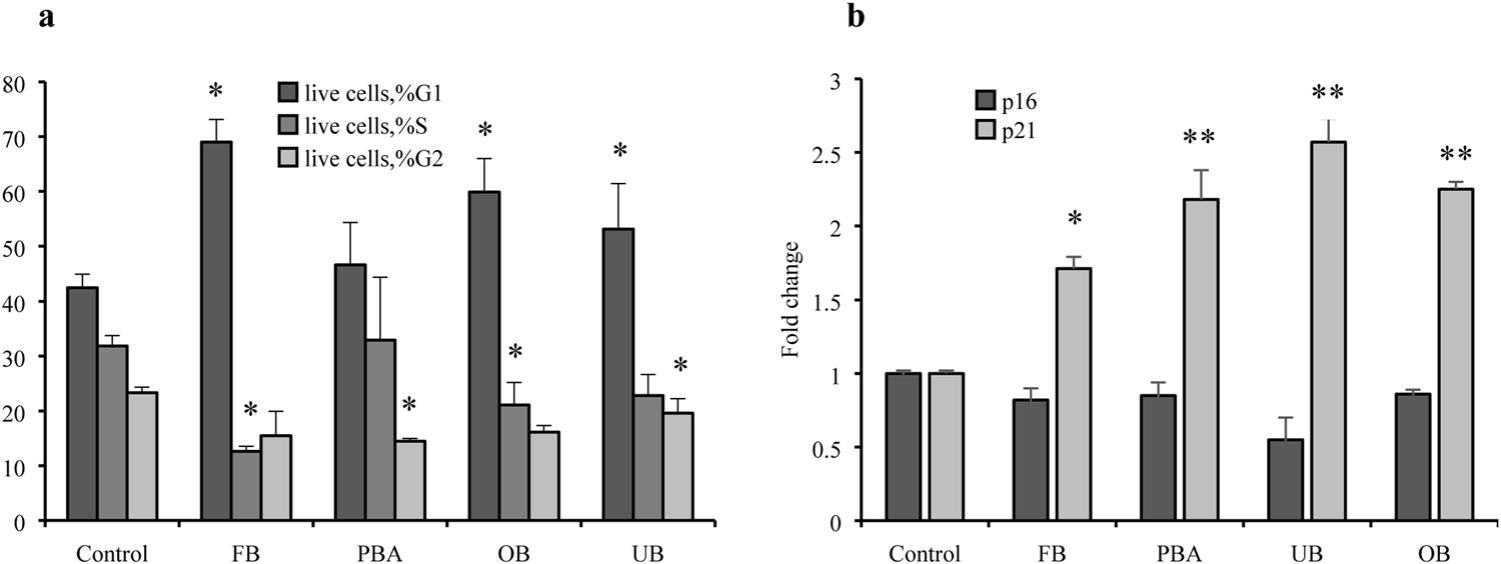
Effect of adhesive extracts on cell cycle (**a**) and related gene expression (**b**). Human gingival fibroblasts were treated with undiluted extracts for 24 h. Cells were collected and stained with PI and analyzed by flow cytometry for percentage of cell in different phases of cell cycle (**a**). p16 and p21 gene expression were evaluated by RT-PCR (**b**). The values represent the mean ± SD of three independent experiments performed in quintuplicate for each dental material. Differences vs. control: *p < 0.05; **p < 0.001

**Fig. 8:**
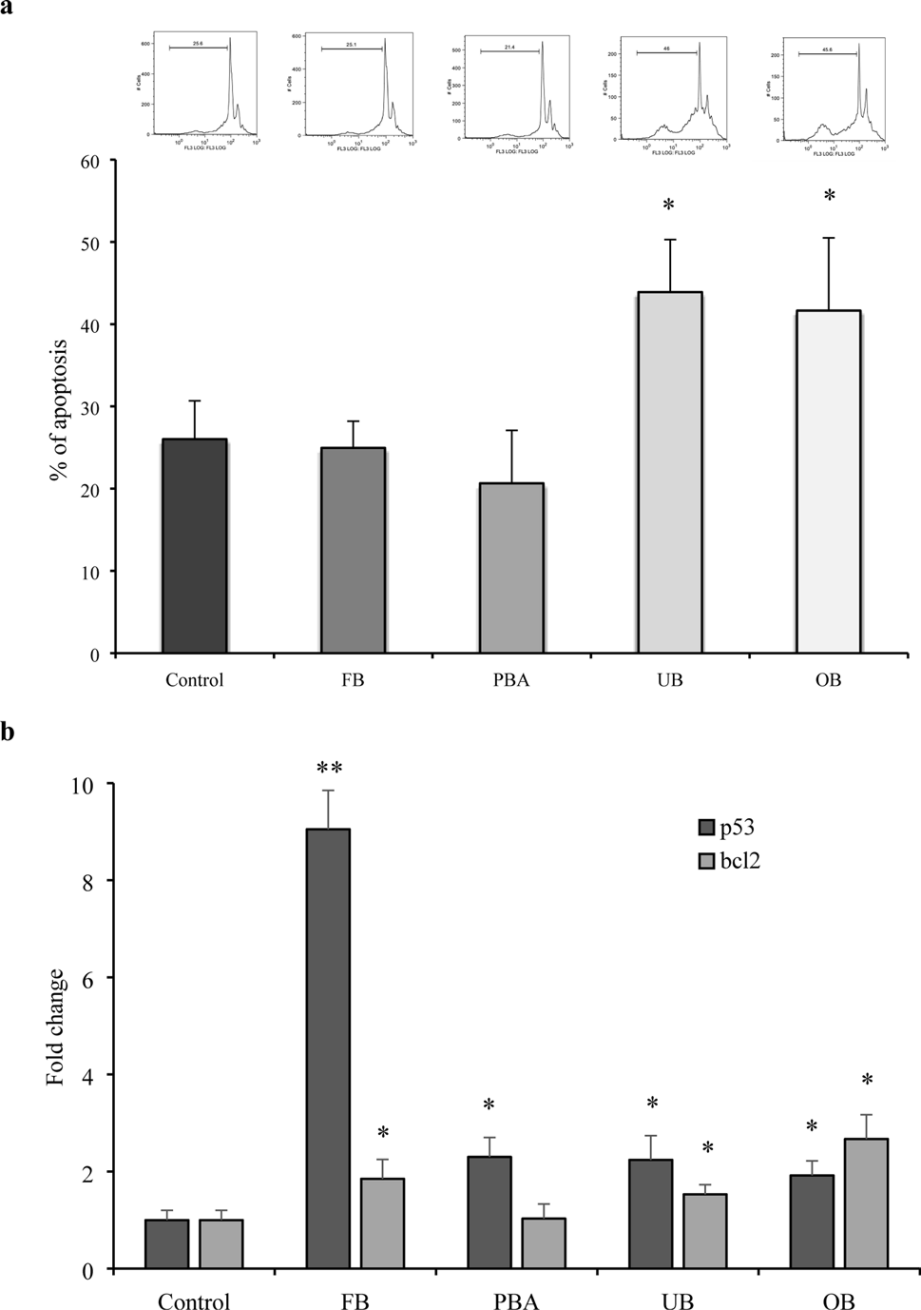
Effect of adhesive extracts on apoptosis (**a**) and related gene expression (**b**). Human gingival fibroblasts were treated with undiluted extracts for 24 h. Cells were collected and stained with PI and analyzed by flow cytometry for percentage of apoptotic cells (**a**). Bcl-2 and p53 gene expression were evaluated by RT-PCR. The values represent the mean ± SD of three independent experiments performed in quintuplicate for each dental material. Differences vs. control: *p < 0.05; **p < 0.001

Compared with controls, FB, OB and UB significantly inhibited fibroblasts in the G0/G1 phase for 24 h, impairing progression to G1–S phase transition. Real-time PCR showed that p16 expression was unchanged, while p21 expression was upregulated at 24 h (Fig. 7). UB and OB upregulated fibroblast apoptosis slightly at 24 h, together with p53 and Bcl-2 expression. PBA did not change apoptosis. It maintained Bcl-2 at control levels and upregulated p53 at 24 h. FB did not modify apoptosis although it increased p53 and Bcl-2 significantly (Fig. 8).

## Discussion

The present study was designed to assess the effects of 4 universal dental adhesives on the adaptative cell responses of human gingival fibroblasts. Contact with the adhesives altered the fibroblast morpho-functional status and migration capacity. Increased ECM protein transcription could indicate tissue evolution towards fibrosis. Interestingly, short-term contact was associated with enzyme stimulation and pro-inflammatory cytokine expression which was followed by a time- and dose-dependent cytotoxic effect. Present results showed that UB and OB impacted most on the human gingival fibroblast morpho-functional profile. In clinical dentistry, knowledge of the potential cytotoxicity of these adhesives is a fundamental requirement for their use [1]. The different PBA and FB response to oral cells, together with the biological knowledge of adhesives behavior, can be useful for clinicians in the material selections and clinical procedures and times.

As far as we are aware, this is the first report of a full range of observations, some of which differ greatly from other studies. In an attempt to dissipate the confusion surrounding such divergent results, the present in-depth analysis established the effects of adhesives on gingival fibroblasts as they constitute the cell type that is most exposed to dental materials [20]. Others instead used murine cells, making comparisons difficult [15, 25]. Negative [23] or positive [19, 21, 25] control systems could also confound comparisons as the present study used only untreated fibroblasts as controls.

Assessing the impact of adhesives at different timepoints could also generate conflicting results. Although observation times generally covered 24 h [15, 23], a few, like the present study extended timepoints to 48 h [8, 22, 25]. In the present study, short- and long-term assessment of cytotoxicity and five dilutions for the MTT test provided better information for both clinicians and researchers, showing cytotoxicity appeared to be dependent on adhesive concentrations and exposure times. In fact, cell viability was first increased and then gradually reduced in a time-dependent manner.

The present study opted to use MTT which assesses cytotoxicity through mitochondrial activity because it is most frequently used in accordance with the ISO 10993-5 recommendations. Some studies assessed different parameters, e.g., the sulforhodamine B SRB assay [20], the lactate dehydrogenase assay (LDH) [14], the fluorescent V-FITC / PI live–dead staining assay [22] or the Hoechst33342 [38].

The MTT assay showed all adhesives stimulated SDH metabolic activity at 1 and 3 h which weakened with longer exposure, thus highlighting damage due to inhibition of normal cellular functions. Morphological analysis and wound healing showed all adhesives induced cell death at 48 h as demonstrated by the numerous round cells in suspension and by the few remaining adherents and prevented cell migration to the wound and wound closure. Further studies are needed to extrapolate these results to the clinical setting.

Although MTT detects cytoxicity, it is non-informative on the mechanism of damage or cell death. In focusing on apoptosis, a common form of cell death, the present study found that unexpectedly, the high cell death rate was not due to apoptosis despite SDH activity, suggesting impaired enzyme function and possibly necrosis. Present observations that p53 and Bcl-2, two key apoptosis-related genes were upregulated compared with untreated cells.[39]. In particular p53 seems to have an important role in the presence of dental monomers like TEGDMA [40]. Interestingly, cell cycle analysis revealed that all the dental adhesives inhibited fibroblasts in the G0/G1 phase and their transition to G1–S, correlating with upregulation of p21, an inhibitor of cell cycle progression at the G1 and S phases. Different studies focused on possible mechanism activated from adhesive monomers, for example, involving ROS production [41, 42].

The major finding in the present study was that all dental adhesives modified inflammatory patterns. Contact with dental materials can, in fact, cause an inflammatory response [43, 44] with over-production of inflammatory markers such as IL6, IL-8 [45, 46], IL1β, IL-18 [47–49], all of which play major roles gingivitis and periodontal destruction [50, 51]. Present observations showed all adhesives were associated with increased IL6 and IL8 expression after short-term exposure which dropped sharply at 48 h and increased IL1β at 48 h. We hypothesize this was due to their secretion in the extracellular compartment which might be an interesting starting point for future studies. In investigating underlying inflammatory pathways, our attention focused on NF-kB and cathepsin B which probably play different roles in regulating expression of inflammation mediators, such as IL1β, IL6 and IL8 [52, 53]. We found that FB and PBA were linked with NF-kB-associated cytokine expression, while the same cannot be said for OB and UB. In these adhesives, where p-NF-kB regulation is lacking, a closer association with the cathepsin B pathway could be hypothesized. This possible different regulation in the inflammatory cytokine expression will be the subject of future studies.

Since inflammation is known to influence ECM organization [54] we monitored the effects of adhesive extracts on transcription of ECM elements.

Specifically, increased fibroblast adhesion as indicated by high fibronectin levels and excess collagen type I, which were observed with all adhesive extracts suggested promotion of fibrosis with consequent gingival tissue impairment [55, 56]. A compensatory mechanism to reduce collagen accumulation was detected in greater transcription of MMP1 collagenase. Interestingly, fibronectin was reported to bind the TLR4 receptor, a member of the receptor family that regulates the NF-kB-dependent synthesis of cytokines [57]. Its link to TLR4 induced an inflammatory response in fibroblasts [58]. Thus increased fibronectin transcription could account for NF-kB activation shortly after treatment with adhesive extracts. Likewise, elastin/elastase trend may underlie a decreased elastic plasticity, which together with the collagen and fibronectin profiles trigger an increase in tissue fibrosis**.**

Starting from the results of our study, the null hypotheses can be rejected.
